# COVID-19 Demonstrates That Inflammation Is a Hyperviscous State

**DOI:** 10.7759/cureus.30603

**Published:** 2022-10-23

**Authors:** Gregory D Sloop, Gheorghe Pop, Joseph J Weidman, John A St. Cyr

**Affiliations:** 1 Pathology, Idaho College of Osteopathic Medicine, Meridian, USA; 2 Cardiology, Radboud University Medical Center, Nijmegen, NLD; 3 Internal Medicine, Independent Researcher, Columbia, USA; 4 Cardiac/Thoracic/Vascular Surgery, Jacqmar, Inc., Minneapolis, USA

**Keywords:** silent hypoxemia, sars-cov-2, innate immunity, fibrinogen, toll-like receptor 8, myocarditis, blood viscosity, covid-19

## Abstract

Many of the complications of severe coronavirus disease-2019 (COVID-19) are caused by blood hyperviscosity driven by marked hyperfibrinogenemia. This results in a distinctive hyperviscosity syndrome which affects areas of high and low shear. A change in blood viscosity causes a threefold inverse change in blood flow, which increases the risk of thrombosis in both arteries and veins despite prophylactic anticoagulation. Increased blood viscosity decreases perfusion of all tissues, including the lungs, heart, and brain. Decreased perfusion of the lungs causes global ventilation-perfusion mismatch which results in silent hypoxemia and decreased efficacy of positive pressure ventilation in treating pulmonary failure in COVID-19. Increased blood viscosity causes a mismatch in oxygen supply and demand in the heart, resulting in myocarditis and ventricular diastolic dysfunction. Decreased perfusion of the brain causes demyelination because of a sublethal cell injury to oligodendrocytes. Hyperviscosity can cause stasis in capillaries, which can cause endothelial necrosis. This can lead to the rarefaction of capillary beds, which is noted in “long-COVID.” The genome of the virus which causes COVID-19, severe acute respiratory syndrome coronavirus 2, contains an extraordinarily high number of the oligonucleotide virulence factor 5’-purine-uridine-uridine-purine-uridine-3’, which binds to toll-like receptor 8, hyperactivating innate immunity. This can lead to a marked elevation in fibrinogen levels and an increased prevalence of neutrophil extracellular traps in pulmonary failure, as seen in COVID-19 patients.

## Introduction and background

One lesson learned from the coronavirus disease 2019 (COVID-19) pandemic is that inflammation is a hyperviscous state. In COVID-19, this hyperviscosity is driven by fibrinogen levels which can be elevated fivefold, higher than in any other commonly diagnosed disease [1]. Because fibrinogen stimulates erythrocyte aggregation in the microvasculature, the clinical manifestations of the hyperviscosity syndrome in COVID-19 differ from the more familiar hyperviscosity syndrome caused by elevated concentrations of monoclonal IgG. This leads to a higher incidence of thrombosis and complications of hypoperfusion in the microcirculation in COVID-19. For this reason, a one centipoise (cP) (centipoise = millipascal-second (mPa.sec)) increase in estimated high shear (high velocity) and low shear (low velocity) blood viscosity were associated with a 36.0% and 7.0% increase in death, respectively (p < 0.001) [vide infra] [2]. These figures suggest that blood viscosity is a much stronger risk factor for death at high shear rates; however, this is not the case because as the shear rate approaches zero, i.e., stasis, blood viscosity asymptotically approaches infinity. Thus, the additional risk of death associated with abnormally high blood viscosity increases sharply at low shear rates, much more than at high shear rates. This is because of reduced tissue perfusion due to progressive erythrocyte aggregation.

Investigations into the cause(s) of this extraordinary hyperfibrinogenemia led to the elucidation of a novel oligonucleotide virulence factor in single-strand RNA viruses, 5’-purine-uridine-uridine-purine-uridine-3’ (purUUpurU), and an insight into the marked cytokine elevation seen in COVID-19, which is sometimes referred to as “cytokine storm syndrome” [3].

## Review

Overview of the complications caused by hyperviscosity

Because of its association with several unexpected, even perplexing complications, the COVID-19 pandemic challenged physicians more than any viral illness since AIDS. Prior to COVID-19, life-threatening viral illnesses such as Ebola virus disease were more commonly associated with hemorrhagic, not thrombotic complications. The large number of cases of COVID-19 revealed the syndrome known as “silent hypoxemia” and the unexpected limitations involving intubation, positive pressure ventilation, and positive end-expiratory pressure in respiratory failure.

The unexpected nature of these complications stemmed from a lack of awareness of the effects of elevated blood viscosity. Because an increase in blood viscosity causes a threefold decrease in blood flow [1], elevated blood viscosity increases the risk of thrombosis and decreases perfusion in the microcirculation of all organs, including the heart, lungs, and brain.

Blood viscosity is elevated in COVID-19

The apparatus to measure blood viscosity is not widely available. Many studies have reported calculated values by using peer-reviewed formulae and common laboratory data such as hematocrit and total plasma protein concentrations. This allows the estimation of blood viscosity in a large population but does not account for abnormalities of erythrocyte deformability and erythrocyte aggregation, both of which are elevated in COVID-19 [4]. Thus, calculated blood viscosity underestimates actual blood viscosity in COVID-19, especially blood viscosity at low shear rates.

High shear blood viscosity is estimated to be increased by 22% and low shear blood viscosity is estimated to be increased by 7% in COVID-19 (n=41) [5]. In a study of 15 patients in an intensive care unit (ICU) in which blood viscosity was measured, not estimated, high shear blood viscosity was 18% higher and low shear viscosity was 19% higher than controls [6]. The fact that the measured low shear blood viscosity was higher than the calculated value demonstrates the importance of erythrocyte aggregation in determining low shear blood viscosity in patients with a tendency for hyperaggregation (the term for erythrocyte aggregation greater than normal) as in COVID-19.

Blood is a non-Newtonian fluid, which means that its viscosity is not constant at different shear rates but rises almost exponentially at low shear rates. This means that a 20% change in shear rate is associated with a much greater change in blood viscosity at low shear values (<10 sec-1) compared to high shear (>50 sec-1) [1].

High shear blood flow occurs in arteries during systole. Low shear blood flow occurs in veins, capillaries, and in certain cases, areas of changing arterial geometry during diastole. The hyperviscosity syndrome seen in COVID-19 is distinctive because it involves both shear domains. The hematocrit affects blood viscosity in both shear domains. 

High shear blood viscosity affects systemic vascular resistance which is sensed by stretch receptors in the left ventricle and initiates the systemic vascular resistance response (SVRR) [7]. The SVRR lowers systemic vascular resistance and blood viscosity in part by reducing the hematocrit via extravascular hemolysis (eryptosis). The SVRR causes the normochromic, normocytic anemia seen in monoclonal gammopathies and is the main reason for anemia in chronic infection [8]. In COVID-19, this hemolysis is one cause of increased plasma levels of lactate dehydrogenase (LDH). Elevated LDH concentrations are associated with a ~6-fold increase in the odds of developing severe COVID-19 and a ~16-fold increase in the odds of death [9]. These data are similar to those which show an increased risk of death with elevated blood viscosity [2] in that both show the pervasive, fundamental pathology caused by increased blood viscosity.

Because the bulk of systemic vascular resistance develops at the level of arterioles, increased low-shear blood viscosity and erythrocyte hyperaggregation are not sensed or corrected by the SVRR. Large molecules can act like glue and foster erythrocyte aggregation by simultaneously binding two erythrocytes. Of these, fibrinogen plays the dominant role in determining the degree of erythrocyte aggregation [10] because it binds to a receptor on the erythrocyte surface, the integrin αvβ3. Once acquired immunity develops several days into a COVID-19 infection, immunoglobins contribute to hyperaggregation.

Erythrocyte aggregation is progressive. Naturally occurring slow flow allows initial aggregation. This slows flow further, allowing more aggregation, which thereafter slows flow even more, and so on. Thus, blood viscosity increases exponentially as the shear rate decreases. In the presence of hyperfibrinogenemia, erythrocyte hyperaggregation can cause stasis in the microvasculature [11]. Capillary endothelial cells depend on the adjacent erythrocytes for oxygen, so prolonged stasis results in capillary necrosis. If capillary stasis becomes widespread, it will decrease tissue perfusion, impair cellular metabolism and potentially alter organ function throughout the body.

Thrombosis

One of the first complications of COVID-19 to receive widespread media attention was thrombosis in relatively young patients in intensive care units (ICU) despite prophylactic anticoagulation. In a registry of 1114 COVID-19 patients, the frequencies of major arterial or venous thromboembolism, major adverse cardiovascular events, and symptomatic venous thromboembolism were 35.3%, 45.9%, and 27.0, respectively [12]. A report of 15 critically ill COVID-19 patients showed that all patients had an elevation in plasma viscosity, ranging from 1.9 to 4.2 cP (normal range: 1.4-1.8 cP). All patients with plasma viscosity > 3.5 cP had thrombosis [13].

Sluggish blood flow predisposes to thrombosis as noted by Virchow in the 19th century. Sluggish blood flow is simply a manifestation of hyperviscosity. Increased blood viscosity subsequently creates larger areas of slower blood flow. This decreases endothelial production of antithrombotic molecules such as prostacyclin and nitric oxide. Sluggish blood flow also decreases the inflow of antithrombotic molecules and reduces the dilution of activated coagulation factors. Elevated blood viscosity is an important contributor to the phenomenon known as “thromboinflammation” [8].

Professor Holger Schmid-Schönbein, a pioneer of hemorheology, likened the risk of thrombosis caused by increased blood viscosity to the accumulation of deadwood in a forest. Preventing a spark decreases the risk of a conflagration, but normalizing the risk requires removing the deadwood. Prophylactic anticoagulation decreases the risk of a thrombotic event, but it remains elevated until blood viscosity returns to normal [1]. Thrombus formation, despite adequate oral anticoagulation, was previously described in a patient with persistent spontaneous echo contrast [14]. Spontaneous echo contrast is the reflection of erythrocyte aggregation, which we described as the consequence of increased blood viscosity [15].

Professor Schmid-Schönbein’s insight may explain the failure of full therapeutic anticoagulation (as opposed to prophylactic anticoagulation) to reduce the need for organ support in critically ill COVID-19 patients, as reported in three trials: Randomized, Embedded, Multi-factorial Adaptive Platform Trial for Community-Acquired Pneumonia (REMAP-CAP), Accelerating COVID-19 Therapeutic Interventions and Vaccines-4 (ACTIV-4), and Antithrombotic Therapy to Ameliorate Complications of COVID-19 (ATTACC) [16]. The failure of full anticoagulation to reduce the need for organ support such as mechanical ventilation, renal dialysis, or drugs to support blood pressure could be due to either continuing thrombosis or decreased tissue perfusion due to hyperviscosity.

Pulmonary complications of COVID-19 caused by hyperviscosity

Decreased Pulmonary Blood Flow

Blood viscosity was shown to be inversely related to pulmonary blood flow in smokers, ex-smokers, and nonsmokers. The authors of that report wrote that blood viscosity is a “significant and forgotten factor that plays an important role in pulmonary and cardiovascular diseases” [17]. The COVID-19 pandemic further demonstrated the negative impact of increased blood viscosity on pulmonary blood flow. Oxygenation is limited by pulmonary blood flow. Unlike the case with alveolar filling processes, positive pressure ventilation is unhelpful in hypoxia caused by reduced pulmonary blood flow. In diseases such as acute respiratory distress syndrome (ARDS), pneumonia, and pulmonary edema, positive pressure drives oxygen from airspaces into alveolar capillaries, thereby improving hypoxia.

However, positive intra-alveolar pressure worsens pulmonary blood flow by increasing right ventricular afterload and decreasing pulmonary venous return, which is facilitated by negative intrathoracic pressure. Failure of positive pressure ventilation to improve oxygenation in many cases of pulmonary failure associated with COVID-19 eventually led to the abandonment of early intubation.

Perhaps the most striking unexpected complication of COVID-19 was “silent hypoxemia,” also called “silent hypoxia” or “happy hypoxia.” It is loosely defined as low oxygen saturation without dyspnea. Oxygen saturations as low as 50% have been reported [18]. This syndrome was mysterious because it occurred in the setting of normal lung compliance. Decreased pulmonary compliance is a nonspecific finding and results from the accumulation of fluid, exudate, or collagen in the lungs. 

Silent hypoxemia is also caused by increased blood viscosity and decreased pulmonary blood flow. Decreased lung perfusion decreases gas exchange, increasing the partial pressure of oxygen (PaO_2_) and reducing the partial pressure of carbon dioxide in alveoli. Because PaO_2_ is already increased, the effect of supplemental oxygen on improving oxygenation is less dramatic than in alveolar filling processes. In COVID-19, increased blood viscosity decreases pulmonary blood flow and causes global ventilation-perfusion (V/Q) mismatch. In COVID-19, hypoxemia due to decreased pulmonary blood flow often precedes the development of parenchymal abnormalities and clinical deterioration [19], again demonstrating the pervasive pathologic effect of increased blood viscosity. 

The absence of dyspnea in association with silent hypoxemia caused physicians to question the accuracy of pulse oximetry. The pulmonary parenchymal (as opposed to the pleura or chest wall) impetus for the sensation of dyspnea is thought to be the firing of perivascular stretch receptors in the pulmonary microvasculature. This is caused by increased, not decreased pulmonary blood flow [20], explaining why dyspnea is not part of the presentation of silent hypoxemia.

Probably the only antecedent of silent hypoxemia was untreated polycythemia vera. In the early twentieth century, Osler described the symptoms of polycythemia vera as cyanosis in winter, incapacity for work, headache, giddiness, dizziness, and disturbance of vision. These symptoms are easily attributable to elevated blood viscosity and hypoxia. Dyspnea is not mentioned. Regarding therapy, Osler wrote, “When there is much fullness of the head and vertigo, repeated bleedings have given relief. Inhalation of oxygen may be tried when the cyanosis is extreme.” The last statement speaks to the decreased efficacy of supplemental oxygen when pulmonary blood flow is decreased because of hyperviscosity.

Pulmonary Microvascular Thrombosis

Elevated blood viscosity at low shear rates contributes to the extensive thrombosis and intussusception of the pulmonary microvasculature noted at the autopsy of COVID-19 patients who died of pulmonary failure [21]. This syndrome is referred to as pulmonary microvascular thrombosis, pulmonary intravascular coagulopathy, and microvascular COVID-19 lung vessels obstructive thromboinflammatory syndrome (microCLOTS). 

The excessive activation of toll-like receptor 8 (TLR8) in COVID-19 **[**vide infra**]** appears to play an important role in pulmonary microvascular thrombosis. Activation of TLR8 in neutrophils leads to furin-dependent proteolytic cleavage of the N-terminal of the Fc gamma receptor IIA (FcgRIIA). This shifts their activity from phagocytosis of immune complexes toward the development of neutrophil extracellular traps (NETs) [22]. NETs are net-like intravascular structures composed of neutrophil chromatin and granule contents. Their function may be to entrap circulating bacteria. By necessity, NETs develop in low shear, as high shear conditions would destroy them like a spider web in the wind. For this reason, NETs formation is accentuated in COVID-19. NETs can reach a diameter of tens of microns, much larger than a capillary, which could embolize into the pulmonary microvasculature. NETs formation is said to be another aspect of the immune response which is “dysregulated” in severe COVID-19 [23].

NETs foster a localized prothrombotic environment because enzymes released from neutrophils inactivate anti-coagulant proteins such as antithrombin, thrombomodulin, protein C, and tissue factor pathway inhibitor [24]. Further, platelets interact with NETs in COVID-19 [23]. Thus, NETs are nidi for microvascular thrombosis. The greater copy number of the oligonucleotide virulence factor 5’-purine-uridine-uridine-purine-uridine-3’ (purUUpurU) in the genome of severe acute respiratory syndrome coronavirus 2 (SARS-CoV-2) compared to influenza A virus [3] **[**vide infr**a]** accounts for the lower incidence of pulmonary microvascular thrombosis in patients who died of pulmonary failure due to influenza [22]. 

Cardiac Complications

In a series of 100 consecutive echocardiograms performed on hospitalized COVID-19 patients, the most common abnormality was right ventricular dilatation/dysfunction, present in 39% of the patients [25]. This finding was associated with a shortened acceleration time, an indicator of increased pulmonary artery resistance, consistent with elevated blood viscosity. Clinical deterioration was associated with further shortening of the acceleration time and worsening right ventricular dilatation, suggesting increased pulmonary vascular resistance. This presentation can be seen with pulmonary embolism, positive pressure ventilation, worsening blood viscosity or a combination of these.

Cardiac diastolic dysfunction was the second most common cardiac abnormality in the aforementioned study, observed in 16% of hospitalized COVID-19 patients [25]. Relaxation of actin-myosin cross-bridges in cardiac muscle, and thus ventricular relaxation after systolic contraction, requires adenosine triphosphate (ATP). The principle is the same as in rigor mortis, in which limbs become fixed in position in the hours after death as ATP is depleted. Thus, decreased tissue perfusion and reduced delivery of energy substrates can lead to diastolic dysfunction.

Although myocarditis has received more attention as a complication of immunization for SARS-CoV-2, the incidence in COVID-19 is much higher. In a retrospective study of 367 consecutive COVID-19-positive adults, the incidence of myocardial injury, defined as a high sensitivity cardiac troponin T (hs-cTnT) concentration above the sex-specific 99th percentile, was 46% [1]. The incidence of myocarditis after immunization is only 0.5 to 2.13 cases per 100,000 immunizations [26].

Myocarditis in both settings is due to blood hyperviscosity which decreases the delivery of oxygen and energy substrates and increases myocardial work by increasing vascular resistance. If the mismatch of myocardial oxygen supply and demand is sufficiently severe, a type 2 myocardial infarction will result. Lesser degrees will cause focal cardiomyocyte necrosis, resulting in histopathologic, biochemical, and radiographic evidence of myocarditis.

Acute myocardial infarction (MI) is a complication of all severe infections, including COVID-19. Like thrombosis in deep veins, this is easily attributable to blood hyperviscosity. The incidence of MI in COVID-19 patients in an ICU is approximately 8%, which is similar to the incidence of inpatients with pneumococcal pneumonia [1,8]. Organization of coronary mural thrombi results in lesions that are indistinguishable from atherosclerotic plaques, resulting in an increased risk of MI which extends 10 years following severe pneumococcal pneumonia [8]. Similarly, survivors of COVID-19 may have a long-term increase in the risk of MI.

Decreased Perfusion of the Central Nervous System

Blood hyperviscosity and decreased perfusion impair the activity of all tissues. Decreased perfusion of the kidneys can cause pre-renal azotemia. Decreased perfusion of the liver can cause hepatocyte damage and elevation of transaminases. Decreased cerebral perfusion can be detected on susceptibility-weighted imaging (SWI), an imaging sequence formerly called “blood oxygen level dependent” (BOLD) venography because it is sensitive to deoxyhemoglobin. In adults with COVID-19, the most common magnetic resonance imaging (MRI) abnormalities of the brain, usually involving white matter, were seen on SWI in 29 of 39 examinations. Overall, white matter lesions were seen in 23 of 41 MRI examinations. The most common indication for performing an MRI of the brain was unexplained prolonged impaired consciousness after extubation. According to the authors, possible pathophysiologic mechanisms for these white matter lesions are hypoxia, ischemia, and stasis of deoxyhemoglobin-rich blood, all of which can be manifestations of blood hyperviscosity [27].

White matter lesions result from decreased myelination of axons by oligodendrocytes. These cells extend myelin-containing cytoplasmic processes around as many as 50 axons. They predominantly use aerobic glycolysis to generate ATP and precursors for myelin synthesis. Hypoxia decreases oligodendrocyte ATP production and the number of cytoplasmic processes, resulting in demyelination and encephalopathy [28]. Thus, demyelination following prolonged hypoperfusion may be reversible, a milder form of delayed post-hypoxic leukoencephalopathy (DPHL). In contrast to DPHL, in which oligodendrocytes suffer lethal injury following a major hypoxic event, prolonged hypoperfusion may cause reversible cell injury and temporary demyelination. 

Long COVID 

Besides being a hyperviscous state, inflammation is also a hypermetabolic and catabolic state. Proteins, particularly actin and myosin, are catabolized for the generation of energy. At the same time, anorexia decreases oral intake of food. The elevated concentrations of tumor necrosis factor (TNF) and interleukin 1 (IL-1) seen in severe COVID-19 accentuate these phenomena.

Hyperviscosity may play a role in the pathogenesis of “long covid.” The decreased perfusion in the microcirculation resulting from hyperviscosity can be viewed as a blockade or siege in wartime. These reduce imports, disrupt the economy, and allow the deterioration of infrastructure. Analogously, hyperviscosity causes widespread sublethal injury. Catabolized and senescent proteins must be replaced, and deteriorated cell membranes also need to be repaired. In the central nervous system, recovery requires the regeneration of neuronal and glial cytoplasm and plasma membrane as well as the removal of debris by microglia.

Thus, one aspect of long covid could be recovery from widespread sublethal cell injury caused by systemic hypoperfusion in the microcirculation due to hyperviscosity [4]. The blockage of the capillary network by the sluggish blood flow will ultimately diminish the capillary density in different tissues, which will permanently affect tissue perfusion, even after the acute inflammatory aspects of the COVID-19 infection have disappeared. This persistent capillary rarefaction has been demonstrated recently by Osiaevi et al. in patients with long COVID syndrome [29].

PurUUpurU hyperactivates innate immunity in COVID-19

We have putatively identified the oligonucleotide purUUpurU as a virulence factor in single-strand RNA (ssRNA) viruses including SARS-CoV-2 [30]. In vitro data of TLR8-bearing myeloid cells incubated with oligonucleotide precursors to purUUpurU demonstrates upregulation of pro-inflammatory cytokines [3]. This oligonucleotide is generated by the breakdown of phagocytosed viruses by host endonucleases within lysosomes. It activates innate immunity by binding to TLR8, resulting in upregulation in the expression of proinflammatory cytokines. Single-strand RNA viruses vary in their genomic purUUpurU content. The pathogenic coronaviruses possess numerous copies [3].

We believe the large number of purUUpurU in SARS-CoV-2 contributes to the markedly elevated levels of proinflammatory cytokines including IL-1, TNF, and interleukin 6 (IL-6) in severe COVID-19. These elevations have drawn comparisons to the “cytokine storm syndrome” seen in certain inflammatory states.

In COVID-19, purUUpurU drives upregulation of IL-6 expression which results in a marked acute phase reaction. In this program, the expression of several large proteins including fibrinogen is increased. This contributes to the extraordinary elevations of fibrinogen and hyperviscosity in COVID-19. Increased activation of TLR8 also favors the formation of NETs [23], resulting in the high incidence of pulmonary microvascular thrombosis at autopsy in COVID-19 patients who died of pulmonary failure [22].

Elucidation of purUUpurU as a virulence factor has provided insight into the pathogenesis of nonspecific viral syndromes. In general, by driving cytokine expression, the copy number of purUUpurU in their genome correlates with the severity of acute illness caused by ssRNA viruses. PurUUpurU is one driver of the symptoms seen in nonspecific viral syndromes and the prodromal phase of many specific viral diseases [30].

It has also provided insight into the cause of the marked elevations of cytokines seen in coronaviral and ebolavirus infections. These elevations may be driven as much, or even more, by the viral genome as by loss of control of the host immune response [30]. 

## Conclusions

COVID-19 has demonstrated that inflammation is a hyperviscous state. Marked hyperviscosity causes many of the symptoms seen in severe COVID-19. Hyperviscosity causes areas of sluggish blood flow, leading to thrombosis. Thus, elevated blood viscosity contributes to “thromboinflammation.” Blood viscosity is inversely related to blood flow. Elevated blood viscosity reduces blood flow to all organs. Decreased pulmonary blood flow causes hypoxia which does not respond as favorably to positive pressure ventilation and supplemental oxygen as do alveolar filling processes such as ARDS, pneumonia, and pulmonary edema. Hyperviscosity also fosters pulmonary microvascular thrombosis. Increased pulmonary vascular resistance caused by hyperviscosity leads to right ventricular dilatation. Decreased myocardial perfusion causes diastolic dysfunction and myocarditis. Hyperviscosity increases the risk of myocardial infarction in all severe infections, including COVID-19. Decreased perfusion of the central nervous system is observed on neuroimaging as demyelination because of sublethal oligodendrocyte injury. Widespread sublethal cell injury and rarefaction of capillary beds may contribute to long COVID.
